# Development and Validation of Knowledge, Attitude, and Practice Questionnaire: Toward Safe Working in Confined Spaces

**DOI:** 10.3390/ijerph19031242

**Published:** 2022-01-22

**Authors:** Hamiza Ngah, Suhaily Mohd Hairon, Nurul Ainun Hamzah, Shahronizam Noordin, Mohd Nazri Shafei

**Affiliations:** 1 Department of Community Medicine, School of Medical Sciences, Universiti Sains Malaysia, Kubang Kerian 16150, Kelantan, Malaysia; mizunithiv@gmail.com (H.N.); suhailymh@usm.my (S.M.H.); 2 Environmental and Occupational Health Programme, School of Health Sciences, Universiti Sains Malaysia, Kubang Kerian 16150, Kelantan, Malaysia; nurulainun@usm.my; 3 National Institute of Occupational Safety and Health, Bangi 43650, Selangor, Malaysia; shahronizam@niosh.com.my

**Keywords:** validation, item response theory, exploratory factor analysis, confirmatory factor analysis, Cronbach’s alpha

## Abstract

Confined space workers do a wide range of tasks, many of which have a significant risk of hazardous exposure. Hence, a reliable and valid questionnaire is important in assessing the knowledge, attitude, and practice (KAP) of workers in this field. The present study was conducted to develop and validate a questionnaire that could assess the KAP for safe working in a confined space. The questionnaire went through a development and validation process. The development stage consisted of a literature review, expert’s opinion, and evaluation by experts in the field via cognitive debriefing. The validation stage encompassed exploratory and confirmatory parts to investigate the psychometric properties of the questionnaire. A total of 350 participants were recruited among confined space workers from two oil and gas companies in Malaysia. The two-parameter logistic item response theory (2-PL IRT) analysis was used for the knowledge section. Exploratory factor analysis (EFA) and confirmatory factor analysis (CFA) were used in the attitude and practice sections of the validation stage. The development stage resulted in 30 items for knowledge, attitude, and practice sections. Items in the knowledge section showed an acceptable difficulty and discrimination, as noted during the 2-PL IRT analysis. The EFA resulted in a one-factor model for attitude and practice sections, and contained 18 items, with factor loading > 0.4. The Cronbach’s alpha was 0.804 and 0.917 for attitude and practice sections, respectively. The CFA for attitude and practice sections indicated a good model fitness (Raykov’s rho = 0.814 and 0.912, respectively). All items indicated good reliability and valid psychometrics for determining KAP on safe working in a confined space.

## 1. Introduction

Workers are often exposed to dangerous situations due to the nature of the confined spaces (CS) in their workplaces which can result in injuries or death. According to the law in Malaysia (Industry code of practice for safe working in a confined space, 2010), a CS is defined as a space that is large enough so that the workers can enter and carry out specific tasks, but that the entry and exit of people is limited, thus resulting in an unfavorable environment that contains or produces a hazardous atmosphere [1]. At any time, CS workers may be exposed to an atmosphere containing potentially dangerous amounts of pollutants, oxygen deficits or excess, and the risk of engulfment [2]. Any activity in a confined space increases the airborne diffusion of particles, toxic gases, and other hazardous pollutants [3,4]. To avoid exposing workers to dangerous conditions, everyone who is compelled to enter a confined space for work reasons must follow the specified rules and regulations [5]. Safety management and operations planning are the essential roles of an organization to prevent injuries, illnesses, and fatalities in the general industry [6,7].

In 2016, the 41 pairs of occupational risk factors and health outcomes were projected to be responsible for 1.9 million deaths worldwide. Long working hours were the cause of the most deaths, followed by occupational particulate matter, gases and fumes, and occupational injuries [8]. According to the US Bureau of Labor Statistics, between 2011 and 2018, around 1030 workers died due to fatal occupational injuries that they sustained in CS. The Census of Fatal Occupational Injuries indicated that the number of people who succumbed to their work injuries per year increased from 88 in 2012 to 166 in 2017 [9]. In Malaysia, the data that were retrieved from the Department of Occupational Safety and Health showed that the number of fatal occupational injuries significantly increased from 21.7% to 30.3%, in different sectors from 2013 to 2016 [10]. It was reported that, the primary cause of death was occupational accidents involving CS workers. This could be attributed to an unsafe work environment [11]. A study conducted by Zakaria et al. (2012) also states that an unsafe work environment is the most common cause of workplace accidents among workers at the workplace. They have proved that individual factors such as fatigue and stress, and the nature of the job, such as unsafe acts, machinery and tools, design of workplace, and training procedures, were found to have a direct impact on workplace accidents [12].

According to Heinrich’s domino theory, all accidents occurring in the residences or workplaces were based on a series of events divided into five factors: social circumstances, individual negligence, unsafe acts, accidents, and injuries [13]. Unsafe conditions or activities were the major factors that caused these incidents and had to be remedied [14]. Knowledge about the dangers of working in CS along with a good attitude and practice could help decrease unsafe activities and conditions [15]. Almost 90% of workplace accidents were due to human error, whereas only about 10% were due to unsuitable equipment or environment if there was no good system to support safety management. The level of success in occupational health and safety management is determined by the way organizations turn their systems and procedures into reality [16]. Confined space accidents could occur if workers are unaware of the hazards or possible dangers within or near the space. Workers may not take into account the new hazards and other situations that arise as a result of working in CS [13]. Workers’ attitudes and practices toward safe work were linked to their understanding of the risks associated with working in such a hazardous environment [17]. A study in Sudan found 56% still have a lack of awareness of the concept of CS and the hazards of working. The workers did not even have the pre-entry hazard identification during entry to CS [18].

To understand the level to which the workers implemented a good attitude and safe practices, based on their knowledge during their work, the researchers evaluated their knowledge, attitude, and practice (KAP) using a basic questionnaire [19]. Assessing their KAP levels indicated the competency and ability of the people to prevent, control, and manage safety measures at any workplace [20]. It is vital to guarantee that health and safety issues are examined, planned, organized, regulated, monitored, recorded, audited, and reviewed in a systematic and holistic way in order to produce a safe and healthy working environment [21]. In addition, it can also analyze a target group’s current knowledge, attitude, and practice on a particular topic in order to identify their needs, challenges, and potential barriers prior to planning and implementing an intervention [22]. A valid instrumental tool is essential to determine the suitability of the KAP survey questions to be used and helps researchers guarantee that they are asking questions genuinely measuring the topics or traits it is meant to measure [23]. According to the literature relevant to this subject, evaluating KAP level using psychometric questionnaire is a way to investigate the existing workplace safety culture [24].

Generally, validated measurement tools to assess the KAP studies are conducted in the construction industry or hospitals, based on overall occupational safety and health factors. The most widely used questionnaire to assess safety culture is the Safety Attitude Questionnaire (SAQ). The SAQ is a self-reported psychometric questionnaire designed to measure safety attitudes of healthcare workers [25,26]. Nonetheless, it is not applicable to CS workers. Workers in CS are subjected to specific working circumstances and environmental exposure. Currently, there is a paucity of standardized and validated measurement tools on the psychometric properties of questionnaires measuring KAP with a focus on safe working in CS. However, there was no research identified that attempted to develop and validate a Malay questionnaire on safe working in confined space. This gap in the research impedes the ability to obtain comprehensive information and an evidence-based approach for policymakers to make more effective safe confined space work implementation decisions to prevent and mitigate confined space-related accidents and injuries in the future. Therefore, this research was conducted to develop and validate a Malay questionnaire that included new content, response processes, and internal structure that assessed the KAP on safe working among the CS workers.

## 2. Materials and Methods

The questionnaire was developed and validated in two phases. Phase 1 comprised of the questionnaire development stage, while Phase 2 included the psychometric validation of the questionnaire using the item response theory (IRT), exploratory factor analysis (EFA), and confirmatory factor analysis (CFA).

### 2.1. Phase 1: Questionnaire Development

The Knowledge, Attitude, and Practice on Safe Working in the Confined Space (CS-KAP) questionnaire was structurally developed and specifically designed for assessing the KAP of confined space workers. This questionnaire was designed based on the information stated in the Occupational Safety and Health Act 1994 and the Industry Code of Practice for Safe Working in a Confined Space (ICOP) 2010 [1,27]. This law and code of practice mandated for employers and employees in Malaysia with the goal of offering practical assistance to employers, parties, and employees for safe working in confined spaces. The development stage comprised of item generation, conceptualization, and questionnaire evaluation by a group of experts in the field, as a part of the response process assessment [28,29]. In addition, item generation was also based on a thorough literature review to discover available resources on KAP toward safe working in a CS, as well as to identify relevant items and scales in the existing questionnaire on safe working [7,13,30,31,32] and discussion with the experts. Five experts, who included a public health physician, biostatistician, occupational and environmental health consultant, and a safety management expert, formed the research team. One research team member acted as a coordinator for the communication processes. Serial meetings conducted with the experts to verify all the identified domains. Thereafter, all domains that were seen to be applicable and appropriate to the CS workers were included in the questionnaire. The same research panel assessed the construct of this questionnaire and judged the validity of the content in terms of coverage, relevance, and representativeness [33]. Communication via emails and face-to-face meetings were also conducted until all the members agreed on a provisional definition for every domain. A pre-survey assessment was performed through cognitive debriefing by applying intensive interviewing to assess the understandability and the errors involved in the interpretation of particular questions, recalling the vital information, performing judgments, and editing all answers. For this purpose, 12 CS workers were recruited from the top management of the water services in the Central Region of Malaysia. In accomplishing this step, two methods for cognitive debriefing were used think-aloud and verbal probing [29,34]. Following that, for each comprehensibility and clarity level, all data were calculated for the face validity index [35]. The results of the response process validity were used to generate a revised and finalized version of the questionnaire that suited a self-administered questionnaire. Table 1 presents a summary of the domains, concepts, and response options that were included in the questionnaire.

### 2.2. Phase 2: Validation

#### Study Setting and Participants

A cross-sectional study was conducted from July to September 2020, among the CS workers working in two oil and gas companies in Malaysia. A total of 350 participants were selected for the study (150 for the EFA and 200 for the CFA and the two-parameter logistic item response theory (2-PL IRT) based on the recommendations of Edelen and Reeve (2007) and Kline (2015) [36,37]. All permanent workers who were Malaysian citizens or contract CS workers, aged above 18 years, and understood Bahasa Malaysia were recruited.

First, a briefing regarding the study was conducted for all the participants, and written consent was obtained from the participants who wished to participate in this study. Finally, all the participants were required to answer the questionnaire by themselves, which took about 15–30 min to complete.

### 2.3. Statistical Analysis

The collected data was analyzed using the R software (ver. 4.0.2), with an RStudio environment [38]. All descriptive analyses included proportion, frequency, mean and standard deviation (SD). In addition, the knowledge section was further analyzed using the 2-PL IRT analysis with the “ltm” package (ver. 1.0.0). On the other hand, the attitude and practice sections were assessed using the EFA with the “psych” package and the CFA with the “lavaan” package (ver. 0.5–22).

#### 2.3.1. Item Response Theory

A 2-PL IRT analysis was conducted to determine the difficulty and discrimination level for every item. The range of difficulty at ±3 was considered acceptable, whereas discrimination values between 0.35 and 2.5 were considered good [39,40]. Item characteristic curves (ICCs) were plotted to assess the correlation between the worker’s ability and their probability to accurately respond to the questions. Item information curves (IICs) were plotted to determine the accuracy of the item for measuring a specific ability level. Furthermore, a Test Response Function (TRF) was also plotted to evaluate the predicted score of the respondents at their specific ability levels with the help of the “irtoys” package. Item fit was determined by conducting a chi-squared goodness-of-fit test for each item. The model fit was further evaluated using the root mean square error of approximation (RMSEA) with a RMSEA ≤ 0.08 indicating a good fit. A chi-squared residual of less than 4 indicated a good fit on the two-way margin [41]. Unidimensionality was determined using a modified parallel analysis [42].

#### 2.3.2. Exploratory Factor Analysis

EFA was used to determine the underlying relationship between the measured variables. Univariate normality was tested using histogram. The Kaiser–Meyer–Olkin (KMO) measure of sampling adequacy with a cutoff point of more than 0.7 [43] and Bartlett’s test of sphericity with a *p*-value of less than 0.05 [44] were used to determine the suitability of the data. Eigenvalue of over one and visual inspection of the scree plot were used to determine the number of factors. The principal axis factoring extraction method, with oblimin rotation was applied. Factor loadings of more than 0.4 and communalities of more than 0.25 were considered acceptable. A Cronbach’s alpha coefficient of more than 0.7 was used to consider a good internal consistency.

#### 2.3.3. Confirmatory Factor Analysis

The robust maximum likelihood (MLR) estimation method was used for non-normally distributed data. The model fit was assessed using the different fit indices along with their respective cutoff values, as follows: χ^2^, *p* > 0.05, a comparative fit index (CFI) and Tucker Lewis fit index (TLI) close to or more than 0.95, a RMSEA, and standardized root mean square (SRMR) ≤ 0.08 [45,46]. Localized areas of misfit were analyzed using standardized residuals (SRs) and modification indices (MIs). Based on the specification with MIs of more than 3.84, factor loadings (FLs) of less than 0.4, and SRs of more than |2.58|, some modifications should be made to improve the model fit by eliminating the items. Factor correlations at <0.85 were considered for stipulating that all factors were different from one another. Regarding reliability, Raykov’s rho index of ≥0.7 was considered acceptable.

## 3. Results

### 3.1. Questionnaire Development and Validity

The important domains and items that could be included in the questionnaire were identified after conducting a broad literature review and undergoing discussions with the expert panel. Of the 41 items, 37 satisfied the content validity criteria. A content validity index by more than five experts should be at least 0.83 when accepting or retaining a specific item [47]. A pre-survey assessment of all the selected items was conducted and seven items were eliminated because of their lack of clarity and comprehensibility. The remaining items were clear in their wording and proper terminologies that could be easily understood by the participants were used. Consequently, a total of three major domains (i.e., KAP) and 30 items were relevant and appropriate with regard to the content and construct that was verified during the last development stage. After making all the changes, the final questionnaire included four sections with 49 items (i.e., 19 items for general information, 8 items for knowledge, 10 items for attitude, and 12 items for practice). The general information studied were age, ethnicity, gender, marital status, level of education, smoking status, alcohol status, presence of comorbidities, occupational status, and presence of training, as well as whether have they have heard of ICOP. The knowledge questionnaires were developed in the form of a three-point Likert-scale, i.e., “yes”, “no”, “unsure” answers, which covers the basics of safe CS work, including equipment and hazard-specific aspects. Questions on safe working procedures and health surveillance were included in the attitude section. The section was evaluated using a five-point Likert scale ranging from 5 to 1 (five = strongly agree, four = agree, three = unsure, two = disagree, one = strongly disagree). Questions about CS risk prevention and personal protective equipment (PPE) were addressed in the practices section. A four-point Likert scale ranging from 1 (never) to 4 (always) was used in the section. For safe practice, scores of “4”, “3,” “2,” and “1” were assigned for replies of “always,” “often,” “seldom,” and “never,” respectively.

### 3.2. Characteristics of the Participants

In this study, all selected participants responded. Majority of the respondents were male with Malay ethnicity (96.6%), with a mean age of 32.1 (SD = 9.80) years. More than half had completed their tertiary education level (51.7%), while 50.6% of the respondents were married. Regarding their employment details, the respondents had an average work experience of 8.3 (SD = 7.96) years, and the mean experience of working in CS was 4.5 (SD = 5.62) years. Most of the respondents spent less than one hour daily working in CS (85.1%), with a primary focus on production (36%). A total of 328 (93.7%) workers were trained to work in CS and almost all of them have heard about the ICOP (2010) rules (98.9%). Table 2 presents the characteristics of the participants.

### 3.3. Item Response Theory

Table 3 shows that in the knowledge section, the questions related to safety measures and safe working conditions in the CS displayed good psychometric properties, based on discrimination and difficulty indices. All the items (except K8) showed a difficulty factor value between −3 and +3, which was acceptable; item K8 showed an extremely low difficulty level of −17.40. Based on the expert advice, this item was then eliminated from the model.

On the other hand, item K1 was retained although it showed a difficulty level that was higher than the cutoff value. The goodness-of-fit analysis indicated that only one of the seven items did not show a good fit, with a *p*-value of <0.05; however, it was still retained as its difficulty and discrimination values were within the range.

**Figure 1:**
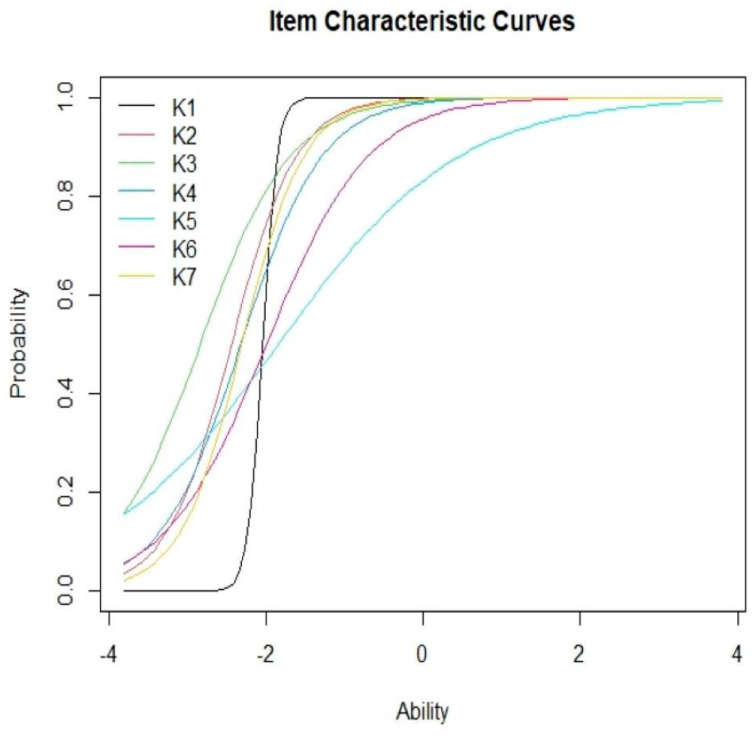
Item-characteristic curves of the test.

**Figure 2:**
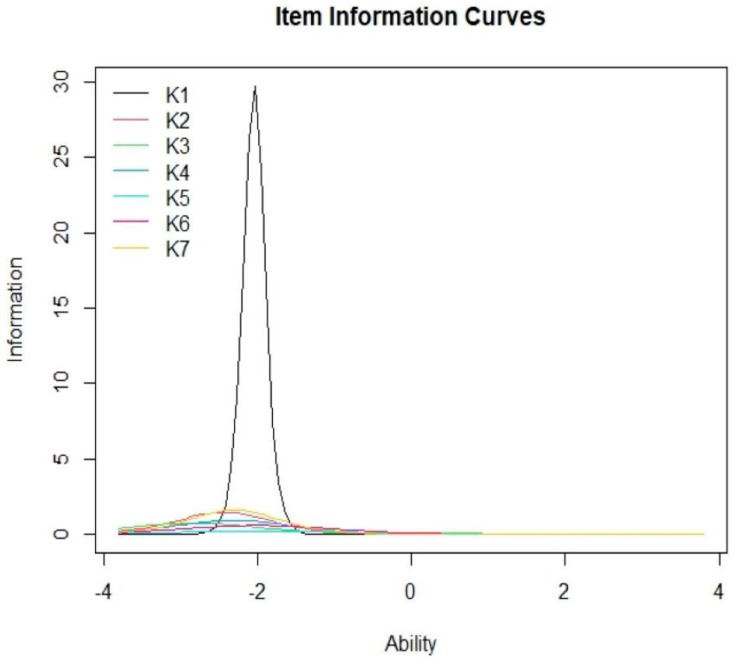
Item- information curves of the test.

**Figure 3:**
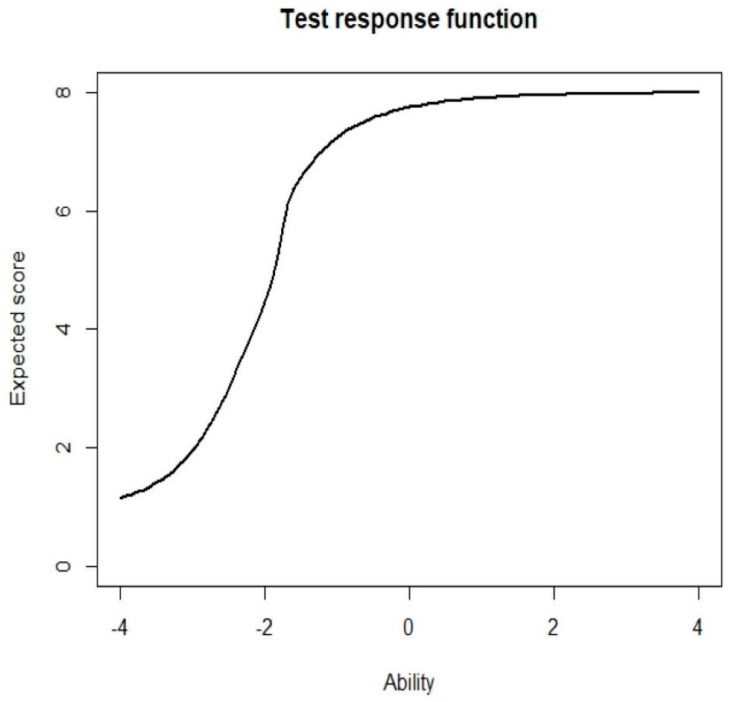
Test- response function of the test.

Figure 1 presents the ICCs for all the items included in the knowledge section. As shown in the figure, IICs indicated that most participants were able to obtain 90–100% correct responses at the +2-ability level for all items. Meanwhile, Figure 2 shows that the maximal item information was derived at the −2.04 K1 ability level, which corresponded to its difficulty level. Figure 3 depicts the TRF that indicated the expected scores for the items with an ability level of 4 were 8 (full mark). The test yielded 88.49% of the amount of information for the −3 to +3 ability range. The ratio of SRs ranged from 0.01 to 2.25, which indicated a good fit for the two-way margins. It was noted that the general data fit well within the model, as the RMSEA value was 0.037, while the scaled CFI was 0.956 and the scaled TLI was 0.938 [48]. The modified parallel analysis proved the unidimensionality assumption of all items (*p* = 0.089). Furthermore, Cronbach’s alpha of 0.631 highlighted the internal consistency between all items.

### 3.4. Exploratory Factor Analysis

Assumption checking was performed for 150 samples with 22 items. Univariate normality was assessed using boxplots, histograms, and Shapiro–Wilk’s tests that showed a *p* < 0.05, indicating a non-normal distribution. Multivariate analysis shows the kurtosis as 75.45 (*p* > 0.05), and dots are away from the line. Hence, the extraction technique of principal axis factoring was used for handling the non-normally distributed data.

The results indicated that the KMO index values were 0.84 and 0.91; with Bartlett’s test of sphericity of <0.001, for the attitude and practice sections, respectively. This indicated that it could be used for factor analysis. Furthermore, the data did not show any multicollinearity problem. Parallel analysis and the use of eigenvalues values for each attitude and practice sections revealed a one-factor model. With regard to the attitude section, six items showed an acceptable factor loading, ranging from 0.46 to 0.82. All other items were eliminated from the defined model as they showed a Cronbach’s alpha of 0.804 (95% confidence interval (CI): 0.759, 0.848). The elimination of the A1, A4, A5, and A7 items increased Cronbach’s alpha by 0.064. The corrected correlation for the items indicated that they had a good correlation with all items. With regard to the practice section, none of the items was removed since all items showed an acceptable communality value (>0.25) and factor loadings (>0.4), as presented in Table 4. The items showed an internal consistency reliability as the Cronbach’s alpha was 0.917 (95% CI: 0.897, 0.937). Furthermore, the corrected item-total correlation indicated that all items displayed a high correlation with the total items.

### 3.5. Confirmatory Factor Analysis

A two-factor model that has good items (i.e., 6 items for the attitude section and 12 items for the practice section) was subjected to CFA. These data did not have a multivariate normal distribution (kurtosis > 5, *p* < 0.05); hence, a robust maximum likelihood (MLR) estimation technique for conducting the CFA was applied. The primary model showed a poor data fit for all the 18 items (χ^2^ [df = 134] = 373.75, *p* < 0.001; SRMR = 0.07; RMSEArobust = 0.11; CFIrobust = 0.84; TLIrobust = 0.82). The revision model was derived after eliminating the items (i.e., P1 and P12) that were having a factor loading of <0.4. Despite these steps, the model fit was not fulfilled; hence, the model was examined further. Five specifications were suggested for MIs > 3.84. The covariance among the items’ residuals (within the factor) were examined and then iteratively added. Two items’ residuals were added to the model: item P2 and item P3, item A3 and item A6. However, the model fit did not fulfil the acceptable threshold value. Thereafter, item A6 was eliminated as it included two SRs with different A3 and P7. The addition of the correlated error (P2 ↔ P3, r = 0.57) significantly improved the final model that showed a good model fitness according to all the fit indices (χ^2^ [df = 88] = 165.51, *p* < 0.001; SRMR = 0.06; RMSEArobust = 0.08; CFIrobust = 0.94; TLIrobust = 0.92). The FLs ranged between 0.449 and 0.880. Furthermore, the attitude and practice factors showed a correlation of r = 0.212. The construct reliability for the attitude and practice sections was based on Raykov’s rho values of 0.814 and 0.921, respectively (Table 4).

## 4. Discussion

To the best of the authors’ knowledge, this is the first study that has explained the process involved in the design and validation of a Malay questionnaire related to safe working conditions in CS. This CS-KAP acts as a simple assessment tool as it was specifically designed and customized for CS workers. It also exhibited an acceptable reliability level and displayed valid psychometrics. This validated questionnaire helped in collecting proper data for answering a research question [49].

The content and face validity results indicated that the respondents could easily interpret all items accurately and that the items represented the constructs of interest. As the content included in the questionnaire must represent the construct [23], some items must be deleted to highlight the psychometric properties of all items [50].

The knowledge section in the CS-KAP displayed acceptable and good psychometric properties that were based on the discrimination and difficulty indices. Since the difficulty factor value was less than 0, it indicated that all items were easy to understand and helped in differentiating the subjects with lower abilities. Item K1 showed a higher cutoff value; however, it was still retained due to the advice of the experts as this item was useful in measuring the knowledge level of the respondents regarding the safety risk assessment. Retaining all the items cannot significantly affect the psychometric properties or reliability of the items. Some of the earlier reports have also retained important items, although they did not fit with the model [50,51]. On the other hand, for item K8, it was not used in the IRT analysis as it showed a low difficulty level. A lower difficulty level reflected that the easy questions could be tested among the respondents as the participants had a higher ability to answer all tested items [52]. It is strongly recommended that the simplest questions be eliminated in the section [53]. It was also found that item K4 did not fit the 2-PL IRT model due to its item-fit statistics. This was attributed to the fact that a small sample population was used in the study (*n* = 150). A large sample size is needed to obtain more accurate results. It is recommended that a sample of more or equal to 500 are needed to estimate the items and test the ability of the respondents [54].

It was found that the results indicated that the unidimensionality of the IRT model was satisfied and that it reflected a single factor that helped in explaining the relationship between all items. This further indicated that these items can be summed together as a total [50]. It was noted that the internal consistency that was determined using the Cronbach’s alpha value for the knowledge section was slightly lower than 0.7, which showed the heterogeneities of these items in the construct. Some of the items (eight items from the knowledge section) could be responsible for the low internal consistency. However, Arslan et al. (2012) stated that the items with a Cronbach’s alpha of equal to 0.6 were acceptable for any research [55,56,57]. In contrast, an earlier study that was conducted among 30 workers in the premises of Manshahr Razy Petrochemical, Iran reported a Cronbach’s alpha value of more than 0.70 for the worker’s knowledge regarding safety and occupational health [58]. However, the description of their validation process in the study was unclear.

The EFA results indicated that the attitude and practice section of the CS-KAP showed good reliability and construct validation. Even the internal structure validity of the attitude section showed a good fit with the one-factor model, which was behavioral-cognitive rather than the proposed three-factor model, as per the tri-partite theory related to affective, behavior, and cognitive [59]. The one-factor model was valid with regard to the thinking-emotion-behavior components that were interrelated with one another. Therefore, the factors were combined based on the correlation between all items and the intentional meaning of all factors, as proposed in the earlier studies. The same process was implemented in the practice section as it was based on a single factor. One factor was selected for every section to minimize overlapping items and acquire a better factor loading. It was found that 6 factors out of the 10 items included in the attitude section showed a factor loading value of more than 0.4. It shows that there is a close relationship between the items and the factors [60]. All items that were included in the practice section showed a factor loading value of more than 0.4. In addition, the attitude and practice sections showed Cronbach’s alpha value of 0.804 and 0.917, respectively, which was similar to those reported earlier [61,62].

The CFA provides convincing evidence that the factor structure is legitimate. In this study, the CS-KAP helped in determining the model fit and assessing the psychometric properties with the help of the CFA. The construct validity that was determined using the CFA indicated that the two-factor models which were attitude and practice domains showed good goodness-of-fit indices after the primary model was modified. The attitude and practice sections in the final model included 5 and 10 items, respectively. These items were selected after comparing the primary and final models based on the Akaike information criterion (AIC) and Bayesian information criterion (BIC). The AIC and BIC values for the primary model were decreased after modification, while the χ2 value was significant, which indicated that the model is fit. The factor loadings for every item ranged between 0.45 and 0.88 and were considered to be acceptable, based on the recommendation by Hair et al. (2010), who stated that values above 0.3 were acceptable [63]. The RMSEA, CFI, and TLI values were seen to be within the intended range. This indicated that the two-factor models have a good fit. RMSEA values of ≤0.6 and CFI and TLI values of ≥0.90 indicated that the model was acceptable. CFA also confirmed the factor structure. The internal consistency reliability indicated by Raykov’s rho value was effective for the practice and attitude sections in the final model.

### Strengths and Limitations

The strength of our study lies on a validated questionnaire that established the fact that a safe working environment in the CS was a vital step in assessing and improving the role played by the competent workers was designed. From the findings, a targeted approach can be implemented which later will decrease the injuries and death rate at the workplace due to a lack of knowledge and unsafe activities and conditions. In addition, a validated CS-KAP questionnaire could be used to determine the needs, problems, and other measures involved in the implementation of a safe environment at the workplace. Furthermore, the questionnaire acts as an assessment tool to determine the regulations or policies that need to be improved for implementing a safe environment at the workplace.

A survey that was conducted among CS workers working in only two oil and gas companies limits the findings of the present study as it could not represent all the CS workers in Malaysia. Further research is needed for validating the CS-KAP among the CS workers in other companies, belonging to other sectors, so that this proposed model could be generalized to include different safe working environments for the CS workers. Furthermore, a large sample population needs to be included to prove the theory that was generated from the EFA and CFA results. The respondents in this study were 100% from male-Malay ethnicity. Therefore, the validity limited to the male-Malays. However, this instrument could also be applied to other ethnicity as well as female and migrant workers as long as they are able to understand Malay language. This instrument was develop based on the Malaysian laws and regulations. Every worker who is mandated to comply with this law are applicable to use this instrument as research tool in assessing KAP on safe working in CS. We proposed that further research should be undertaken involving other industries that employed female and people of other ethnicity to see whether there is a difference between the current study and future studies. On top of that, the design of the questionnaire, which was based on the judgment of the experts, could make it susceptible to imprecise interpretation and a biased understanding. Future studies need to be conducted to determine additional items and include more scope for safe working conditions in CS for generating more valid and reliable results. Despite these limitations, all instruments used were satisfied since the items and instruments used reflected the major component that highlighted the safe working conditions for the CS workers.

## 5. Conclusions

A questionnaire tailored to the population of confined space workers was created for the research, which is being undertaken for the first time in the territory of Malaysia’s central region. A Malay version of CS-KAP questionnaire was developed and validated among oil and gas workers operating in a confined space. The use of IRT, EFA, and CFA model analysis in confirming the reliability and accuracy of each item in the CS-KAP questionnaire was highlighted. The questionnaire consisted of 22 items with 7 items on knowledge, 5 items on attitude, and 10 items on practice sections. All the items included in every section displayed good reliability and valid psychometric properties that could be used in assessing the KAP of safe working conditions in CS. This instrument is applicable in assessing KAP of safe confined space working not only in oil and gas workers, but also across industries such as manufacturing, construction, utilities, and agriculture as well. The use of this questionnaire is also recommended to measure KAP among workers who are exposed to other hazards such as electrical, falls, and ergonomics. The findings of the research serve as the foundation for the development of preventive and educational programs, as well as amendments to the legal standards that govern this field. However, a further questionnaire development on a bigger scope of safe working in confined space is warranted in future studies to develop stronger reliability of the questionnaire.

## Figures and Tables

**Table 1:** CS-KAP questionnaire on safe working in confined space.

Sections	No. of Items	Concepts Measured	Response Options
Proforma (general information)	19	Socio-demographic, job characteristic (working experience (years), job scope), workplace characteristic, training, source of information	Closed-ended, multiple-choice
Knowledge	8 (1 reverse statement)	General requirement on safe working in confined spaces, confined spaces entry program, employee training and safety equipment	True/False/Unsure; 1 = True 2 = False 3 = Unsure
Attitude	10 (1 reverse-scored item)	Measure three components towards safe working in confined spaces based on tri-partite theory (Lawrence, 2008). The following components as below: Cognitive: belief, thought, attributes Affective: emotion/feeling Behavioral: past experiences	Five-Likert scale option 1 = Strongly disagree 2 = Disagree 3 = Unsure 4 = Agree 5 = Strongly agree
Practice	12	Practices on safe working in confined spaces	Four- Likert scale option 1 = Never 2 = Seldom 3 = Often 4 = Always

**Table 2:** Socio-demographic characteristics of confined spaces workers (*n* = 350).

Variables	Mean (SD)	*n* (%)
Age (year)	32.1 (9.80)	
Gender		
Male		337 (96.3)
Female		13 (3.7)
Ethnicity		
Malay		338 (96.6)
vNon-Malay		12 (3.4)
Marital status		
Single		171 (48.8)
Married		177 (50.6)
Widow/widower		2 (0.6)
Educational level		
No formal education		1 (0.3)
Primary		3 (0.9)
Secondary		165 (47.1)
Tertiary		181 (51.7)
Employment		
Total work experience (years)	8.3 (7.96)	
Experience in CS (years)	4.5 (5.62)	
Working hour in CS per day		
≤1		298 (85.1)
2–5		40 (11.5)
>5		12 (3.4)
Job scope		
Cleaning		90 (25.7)
Inspection		24 (6.9)
Maintenance		110 (31.4)
Production		126 (36.0)
Training		
Yes		328 (93.7)
No		22 (6.3)
Have heard on ICOPs		
Yes		346 (98.9)
No		4 (1.1)

**Table 3:** Result of the IRT analysis in the knowledge section in validation study (*n* = 200).

Items after Removal	b (SE)	α (SE)	χ^2^ (df = 8)	*p* Value
K1 Occupational risk assessment (Hazard identification, risk assessment and risk control-HIRARC) must be done before the entry of workers in confined spaces	−2.04 (0.09)	10.89 (34.64)	2.39	0.967
K2 Employers need to ensure that warning signs “DANGER-CONFINED SPACE. NO ENTRY” is placed near the entrance of the confined spaces	−2.44 (0.50)	2.42 (1.18)	10.72	0.213
K3 Confined space workers are exposed to hazardous gases within the scope of the workplace	−2.84 (0.83)	1.74 (0.89)	21.34	0.013
K4 Confined space workers must have confined space entry training recognized by the Department of Occupational Safety and Health	−2.30 (0.47)	1.97 (0.81)	32.67	<0.001
K5 Ventilation in the confined space should be placed at the beginning of the confined space work only when work is carried out	−1.83 (0.59)	0.87 (0.35)	13.26	0.120
K6 Exhaust from any equipment placed near a confined space is the cause of the existence of a hazardous atmosphere in the confined space	−2.00 (0.41)	1.53 (0.53)	56.50	0.008
K7 Difficulty breathing is a sign of exposure to hazardous atmosphere when working in a confined space	−2.31 (0.40)	2.53 (1.15)	22.83	0.062

On assessment of fit for two-way margins, all item pairs showed good fit. Modified parallel analysis supported unidimensionality, RMSEA = 0.037, M2 = 25.347, TLI = 0.938, CFI = 0.956. α discrimination, b difficulty, df degree of freedom, IRT item response theory, SE standard error, χ^2^ chi-square, RMSEA Root Mean Square Error of Approximation, TLI Tucker-Lewis Index, CFI Comparative Fit Index.

**Table 4:** Results of the EFA and CFA of the attitude and practice sections.

Factors	Items	EFA (*n* = 150)		CFA (*n* = 200)	
		λ	Reliability a	λ	Reliability b
Attitude	A2 I believe employees and employers are fully responsible for the safety of employees in the workplace	0.74	0.804	0.70	0.814
	A3 I believe the entry permit to the confined space needs to be informed and explained to the employees before the confined space work is carried out	0.77		0.64	
	A6 I will stop working in confined space if the gas tester level indicator exceeds the set standards	0.46		-	
	A8 I think the health check-ups of confined space workers should be done periodically	0.47		0.45	
	A9 I believe occupational health and safety campaigns are an effective way to promote and educate employees	0.68		0.69	
	A10 Occupational health and safety are my top priority when I do the confined space work	0.82		0.87	
Practice	P1 I will check the confined space work permit before handling work in the confined space	0.49	0.917	-	0.912
	P2 I make sure the situation in the confined space is safe before entering the confined space	0.78		0.72	
	P3 I check all safety equipment and work tools are in a safe condition to use	0.82		0.77	
	P4 I tell the employer if the safety equipment to do the work in the confined space is incomplete	0.79		0.72	
	P5 I wear safety gloves while handling work in confined spaces	0.89		0.88	
	P6 I wear a safety helmet when handling work in a confined space	0.90		0.88	
	P7 I wear eye protection when handling work in a confined space	0.85		0.87	
	P8 I wear ear protection when handling work in a confined space	0.78		0.81	
	P9 I wear respiratory protection while handling work in a confined space	0.88		0.81	
	P10 I wear a body harness while handling work in a confined space	0.57		0.57	
	P11 I wear a reflective safety jacket while handling work in a confined space	0.55		0.60	
	P12 I joined the employer for a feedback session after the end of the confined space entry operation	0.45		-	

EFA expoloratory factor analysis, CFA confirmatory factor analysis, λ factor loading/standardized loading. ^a^ Cronbach’s alpha, ^b^ Raykov’s rho.

## Data Availability

Not applicable.
